# An Overview of Biopolymer-Based Graphene Nanocomposites for Biotechnological Applications

**DOI:** 10.3390/ma18132978

**Published:** 2025-06-23

**Authors:** Roya Binaymotlagh, Laura Chronopoulou, Cleofe Palocci

**Affiliations:** 1 Department of Chemistry, Sapienza University of Rome, Piazzale Aldo Moro 5, 00185 Rome, Italy; roya.binaymotlagh@uniroma1.it (R.B.); laura.chronopoulou@uniroma1.it (L.C.); 2 Research Center for Applied Sciences to the Safeguard of Environment and Cultural Heritage (CIABC), Sapienza University of Rome, Piazzale Aldo Moro 5, 00185 Rome, Italy

**Keywords:** bio-nanocomposites, graphene, graphene oxide, biopolymers, drug delivery, tissue engineering, wound healing, food industry, biotechnological applications

## Abstract

Bio-nanocomposites represent an advanced class of materials that combine the unique properties of nanomaterials with biopolymers, enhancing mechanical, electrical and thermal properties while ensuring biodegradability, biocompatibility and sustainability. These materials are gaining increasing attention, particularly in biomedical applications, due to their ability to interact with biological systems in ways that conventional materials cannot. Graphene and graphene oxide (GO), two of the most well-known nanocarbon-based materials, have garnered substantial interest in bio-nanocomposite research because of their extraordinary properties such as high surface area, excellent electrical conductivity, mechanical strength and biocompatibility. The integration of graphene-based nanomaterials within biopolymers, such as polysaccharides and proteins, forms a new class of bio-nanocomposites that can be tailored for a wide range of biological applications. This review explores the synthesis methods, properties and biotechnological applications of graphene-based bio-nanocomposites, with a particular focus on polysaccharide-based and protein-based composites. Emphasis is placed on the biotechnological potential of these materials, including drug delivery, tissue engineering, wound healing, antimicrobial activities and industrial food applications. Additionally, biodegradable polymers such as polylactic acid, hyaluronic acid and polyethylene glycol, which play a crucial role in biotechnological applications, will be discussed.

## 1. Introduction

The increasing global demand for sustainable and high-performance materials has led to extensive research into biodegradable bio-nanocomposites [1,2,3], particularly those incorporating graphene and its derivatives, such as graphene oxide (GO) [4,5]. These materials are gaining attention for their superior mechanical, thermal and electrical properties, making them suitable for various industrial, biomedical and environmental applications [6,7]. Biopolymers from renewable sources offer significant benefits such as biodegradability and biocompatibility. Graphene-based materials can be biocompatible, but their compatibility depends on factors like surface chemistry, functionalization and concentration. Functionalized graphene, GO and reduced GO (rGO) with modified surfaces—such as hydroxyl, carboxyl and amino groups—tend to improve interactions with biological systems and reduce toxicity.

Several studies have shown that accurately engineered graphene-based materials can be suitable for biomedical applications like drug delivery, tissue engineering and biosensing. However, at high concentrations or in certain forms, graphene can induce oxidative stress or cellular damage. Therefore, researchers continue refining graphene’s properties to ensure its safe use in medical and biotech fields.

However, the intrinsic limitations of biopolymers, including low mechanical strength, inadequate thermal stability and sensitivity to moisture, necessitate reinforcement with nanofillers like GO to enhance their overall performance [8,9].

Graphene, a two-dimensional nanomaterial composed of sp^2^-hybridized carbon atoms arranged in a hexagonal lattice, possesses outstanding strength, conductivity and barrier properties [10,11]. However, its strong van der Waals interactions often lead to aggregation, limiting effective dispersion within polymer matrices [12,13]. To overcome this limitation, graphene is oxidized to form GO, introducing oxygen-containing functional groups that improve its solubility and interfacial adhesion with biopolymers [14,15]. This functionalization makes GO an ideal candidate for reinforcing biodegradable polymer composites [16,17]. Studies indicate that incorporating low amounts of GO into biopolymer matrices can significantly improve their mechanical properties. For example, the tensile strength of poly(lactic acid) (PLA)-GO nanocomposites can be enhanced by over 100% with only 0.5 wt% GO due to strong interfacial interactions and uniform dispersion [18,19].

Beyond mechanical reinforcement, graphene-based bio-nanocomposites demonstrate substantial improvements in thermal stability. Increasing the thermal stability of GO nanomaterials involves modifying their structure and composition to resist degradation at high temperatures. Here are several strategies:

- Reduction of GO—converting GO into rGO removes oxygen-containing functional groups, improving thermal stability.
- Surface functionalization—coating GO with polymers, metal oxides or other stabilizing agents can enhance heat resistance.
- Doping with elements—introducing elements like nitrogen, boron, or phosphorus can strengthen GO’s thermal properties.
- Crosslinking—using crosslinking agents to bond GO sheets together improves structural integrity under heat.
- Composite formation—embedding GO into materials like ceramics or metal matrices can prevent decomposition at high temperatures.
- Controlled synthesis—optimizing oxidation and exfoliation processes can yield more stable GO structures.

The restriction of polymer chain mobility and the formation of a thermally stable interphase contribute to increased decomposition temperatures [20]. Research on PLA-GO nanocomposites has shown that incorporating GO can elevate the thermal degradation temperature by approximately 20 °C, confirming its effectiveness in thermal applications [21]. Additionally, the electrical conductivity of biopolymer–graphene composites experiences significant enhancements, enabling their use in flexible electronics, biosensors and energy storage devices [22,23]. Graphene’s high electron mobility and percolation network facilitate charge transfer, increasing conductivity by several orders of magnitude compared to pristine biopolymers [24,25]. Another key advantage of graphene-based bio-nanocomposites is their ability to improve the barrier properties of biopolymers, making them suitable for packaging applications [26,27]. The incorporation of graphene derivatives reduces the permeability of oxygen and water vapor by creating a tortuous path, thereby extending the shelf life of food and pharmaceutical products [28,29]. For instance, sodium alginate–GO nanocomposites have demonstrated up to a 59% reduction in water vapor permeability at low filler loadings, enhancing their suitability for sustainable packaging solutions [30].

The fabrication methods employed for biopolymer–graphene nanocomposites play a crucial role in determining their final properties. Several processing techniques, including solution intercalation, melt blending, in situ polymerization and electrospinning, have been explored to achieve homogeneous graphene dispersion [31,32]. Among these, solution intercalation remains the most used method due to its simplicity and effectiveness in dispersing graphene derivatives within polymer matrices [28,33]. Recent advancements have focused on optimizing processing parameters, such as ultrasonication time, solvent selection and functionalization strategies, to improve the interfacial adhesion between biopolymers and graphene [34,35].

Despite these advancements, several challenges remain in the large-scale production of biopolymer–graphene nanocomposites, particularly in achieving uniform dispersion and preventing graphene aggregation [36,37]. Additionally, the composite’s environmental impact and biodegradability require further investigation to ensure sustainability and regulatory compliance [38,39]. Developing cost-effective and scalable synthesis methods, coupled with innovative functionalization approaches, is essential for optimizing graphene-based bio-nanocomposite properties and commercial viability [40,41]. Current research often focuses either on polysaccharide or protein matrices independently, while a comprehensive evaluation of their multifunctional biotechnological applications—particularly integrating drug delivery, tissue engineering, antimicrobial activity and food industry potential—remains fragmented. The present review aims to investigate graphene--/GO-based bio-nanocomposites that combine both polysaccharide and protein biopolymers alongside biodegradable synthetic polymers, enabling a versatile platform with enhanced structural, functional and biocompatible properties.

## 2. Graphene and GO: Properties and Synthetic+ Approaches

Graphene and GO synthesis can follow two primary approaches: top-down or bottom-up. The choice of the synthesis technique affects the material’s quality, scalability and cost, making it crucial for specific applications. Top-down approaches are broadly classified into the exfoliation and oxidation of graphite, widely used for obtaining few-layer graphene, which involves peeling layers from bulk graphite using adhesive tape, a technique pioneered by Novoselov et al. [42]. Other fabrication techniques include unzipping multiwall carbon nanotubes (MWCNTs), micromechanical exfoliation and mechanically assisted exfoliation combined with ultrasonication [43]. Additionally, graphene can be grown on crystalline silicon carbide substrates to achieve high-quality layers suitable for advanced applications. In mechanical exfoliation, external forces—such as stress applied through the scotch tape, atomic force microscopy (AFM) tips, electric fields or sonication—separate graphene layers from graphite [44,45]. Chemical exfoliation, on the other hand, employs alkali metal intercalation to weaken the van der Waals forces between layers, followed by liquid-phase sonication to produce single-layer graphene [46,47]. Another common route is chemical synthesis, where GO undergoes reduction to yield graphene sheets in large quantities [48,49]. This method, often used for mass production, enables the scalable fabrication of graphene-based materials. GO reduction using different reducing agents is significantly affected by molar ratios, temperature and reaction time. Each of these synthesis techniques plays a vital role in tailoring graphene properties for applications ranging from nanoelectronics to biocomposites.

The oxidation of graphite to produce GO has been developed through several established methods, including those introduced by Brodie, Staudenmaier, Offeman and Hummers [50,51]. These techniques rely on the use of strong acids and oxidizing agents to facilitate the oxidation process [52]. The extent of oxidation varies based on the chosen method, which influences the reaction conditions and the properties of the resulting GO [53,54]. Hummers’ method, a widely adopted approach, employs potassium permanganate (KMnO_4_) and concentrated sulfuric acid (H_2_SO_4_) to oxidize graphite, a process not only known for its efficiency but that is also associated with toxic byproducts [55]. The oxidation process introduces functional groups such as epoxy, hydroxyl and carboxylic groups onto the graphene structure, altering its chemical properties and increasing its hydrophilicity [56].

Unlike top-down techniques, bottom-up methods build graphene sheets from individual carbon atoms or molecules, allowing for greater precision in controlling their properties [8]. These techniques include pyrolysis, epitaxial growth on silicon carbide (SiC), chemical vapor deposition (CVD) and other emerging fabrication approaches [56]. Among these, CVD and epitaxial growth have gained prominence due to their capability to produce high-quality, large-area graphene layers [57]. CVD involves breaking down hydrocarbon gases at high temperatures in the presence of a catalyst, leading to graphene formation on a substrate [58]. The choice of the catalyst material and processing conditions greatly influences the structural characteristics and crystallinity of the final graphene layer [59]. Pyrolysis, in contrast, synthesizes graphene by thermally decomposing organic materials in a controlled environment [60]. The process is typically carried out under high pressure to regulate the carbon-to-oxygen ratio, which plays a key role in defining the electrical and mechanical performance of the material [61]. Additionally, ultrasonication methods have been employed to assist in exfoliating graphene layers from sodium ethoxide solutions, leading to improved yield and structural integrity [42].

Epitaxial growth relies on the thermal decomposition of SiC, during which silicon atoms evaporate, leaving behind a graphene layer on the substrate surface [62]. The quality of the graphene formed through this method depends on several parameters, including the orientation of the SiC crystal, the annealing temperature and processing times [63]. This method is especially favored in electronic applications because it produces graphene with high electron mobility and a well-ordered atomic structure [64]. Table 1 summarizes the synthetic methods described, highlighting their advantages and disadvantages.

## 3. Preparation of Graphene-/GO-Based Nanocomposites

To fabricate a stable biopolymer–graphene/GO bio-nanocomposite, achieving a good distribution of carbon-based materials within polymeric matrices is essential [8]. Proper dispersion influences various properties, including mechanical, thermal, electrical, water vapor permeability and gas-barrier performance [65]. A major challenge in this field is the aggregation, incomplete exfoliation and restacking of graphene or GO particles when incorporated into biopolymers, which can negatively affect the characteristics of nanocomposites [66]. The aggregation of GO nanocomposites can be a challenge. Still, several strategies can help improve dispersion and stability, such as surface functionalization and covalent modification with carboxyl, amine or hydroxyl groups, which enhance solubility and prevent aggregation. Non-covalent interactions, which use surfactants, polymers or biomolecules for stabilizing GO sheets, pH and electrostatic control, and ultrasonication can also be effective methods to prevent aggregation.

GO offers additional advantages for processing, as it can be exfoliated in water, making it easier to fabricate nanocomposites with water-soluble polymers, like poly(vinyl alcohol) (PVA) and poly(ethylene oxide) (PEO), or using water-based latex technology [67].

Several processes are used to incorporate graphene and GO fillers into polymer matrices, such as solution intercalation, melt intercalation and in situ polymerization, which are the three main methods [68]. Among these, solution intercalation is one of the most common methods [69]. This involves mixing colloidal suspensions of graphene or GO with suitable polymers, followed by solvent evaporation [70]. The polymer then reassembles around the filler, forming a sandwich structure and creating the bio-nanocomposite [71]. This method has been applied to bio-nanocomposites with materials such as starch, chitosan, PLA, cellulose, alginate, gelatin and poly(hydroxyalkanoates) [72]. The chemical structure of the polymer may change slightly with an increasing amount of graphene or GO, suggesting that both physical interactions (like hydrogen bonding) and minor chemical reactions occur between the biopolymers and the fillers [73]. Solution-based methods can also be used with non-water-soluble polymers like poly(methyl methacrylate) (PMMA) and polyurethanes by chemically modifying GO [74]. In situ polymerization is another effective method, which involves solvents to reduce dispersion viscosity, facilitating the incorporation of fillers [75]. For example, the intercalative polymerization of methyl methacrylate and epoxy resins with GO has been used to prepare nanocomposites with enhanced properties [76]. This method has been successfully applied to create polyethylene and polypropylene/GO nanocomposites [77]. Grafting PMMA chains onto GO also improves the compatibility between the filler and the polymer matrix [78]. Melt blending is a widely used process to disperse particles into polymeric matrices [79]. It has been applied to disperse thermally reduced GO in polymers like polycarbonate and poly(ethylene-2,6-naphthalate) and to distribute expanded graphene or GO into renewable PLA [80]. While this process achieves favorable distribution, it can increase the viscosity of the polymer melt, complicating the procedure [81]. Another method, solid-state shear dispersion, uses a modified twin-screw extruder to prepare polymer composites by dispersing unmodified graphite into polypropylene [82,83].

Besides graphene or GO, carbon nanotubes (CNTs) are also promising for reinforcement, though they require chemical modification due to their tendency to aggregate [83]. While GO aggregation is not a major issue, incomplete exfoliation and restacking are common [84]. The mechanism for interaction in polymer–graphene nanocomposites is influenced by factors such as polarity, molecular weight, hydrophobicity and the nature of reactive groups in the polymer, graphene and solvent [85]. Apart from the main methods, there are additional methods to prepare graphene/GO bio-nanocomposites, as reported in Table 2.

## 4. Biopolymers in Graphene-/GO-Based Nanocomposites

The use of biopolymers in combination with graphene and GO has become a rapidly growing area of research aimed at creating high-performance, eco-friendly materials that, however, often suffer from limited mechanical and thermal properties [87,88]. Introducing graphene or GO into biopolymer matrices provides a means to overcome these limitations. Thanks to their exceptional strength, electrical conductivity and unique two-dimensional structure, graphene-based fillers improve the overall functionality of biopolymer composites. The oxygen-containing groups present in GO also facilitate better compatibility and dispersion within hydrophilic biopolymer systems, enhancing interfacial bonding [89]. The investigation into biopolymer–graphene nanocomposites continues to progress rapidly, emphasizing enhanced filler distribution, stronger matrix–filler interactions and scalable manufacturing processes. Through strategic material choices and refined processing techniques, these composites offer promising opportunities for durable, eco-friendly solutions in a wide range of fields. The upcoming sections will explore their diverse applications in detail.

For example, Fonseca et al. [88] designed a novel nanocomposite incorporating GO and mesoporous amino silica nanoparticles (MSNs). In this composite, MSNs were covalently bonded to the GO surface through an amidation reaction, forming amine–epoxy bonds. This process unexpectedly reduced GO via interactions with H_2_N-MSNs. The synthesis of GO-MSN nanocomposites involved attaching MSNs onto GO sheets using crosslinking agents, enabling the formation of amide bonds.

Research on biopolymer–graphene nanocomposites remains highly active, with a focus on improving dispersion, interfacial bonding and large-scale production techniques [90]. By carefully selecting materials and optimizing fabrication methods, these nanocomposites show immense potential for sustainable, high-performance applications across various industries, which will be discussed in the following sections.

### 4.1. Polysaccharide-Based Graphene Bio-Nanocomposites

Polysaccharides derived from renewable sources like algae, plants, fungi and bacteria are essential macromolecular polymers in biological systems. They contribute to cell communication, adhesion and immune recognition [91]. Their integration with graphene-based materials enhances water dispersibility and biodegradability, making them valuable for biomedical and environmental applications [92]. In this context, we aim to explore various polysaccharides that have been employed to form bio-composites with graphene and GO, highlighting their unique properties and potential uses.

*Starch,* a biodegradable, renewable and cost-effective biopolymer obtained from plant sources, has attracted considerable attention for sustainable material development, especially in fields such as food packaging, biomedical applications and agriculture. However, its practical use is restricted by inherent drawbacks, including high hydrophilicity and relatively poor mechanical and thermal stability. Incorporating GO into starch matrices has proven to be effective overcoming these challenges. The oxygen-containing functional groups present on the GO surface facilitate strong interactions with starch, leading to significant improvements in mechanical strength, thermal stability and barrier performance. This enhanced compatibility between starch and GO nanofillers results in biocomposites with properties better suited for different applications [93].

Polypiperazinamide membranes are widely used for nanofiltration but suffer from low flux due to their flexible hydrophobic backbone. To enhance permeability, GO was incorporated into the top layer of these membranes. However, given the high cost of GO, it was modified with starch, an inexpensive and environmentally friendly hydrophilic matrix, to reduce production costs. A high-flux hyperbranched starch-functionalized GO composite (HGOST) nanofiltration membrane was developed by integrating starch-functionalized GO within the polypiperazinamide network. GO–starch composites were incorporated into the polyamide (PA) top layer via esterification, leading to a reduction in the contact angle to 22.18° due to the abundant oxygen functional groups in both starch and GO. With the incorporation of HGOST, membrane permeance increased to 79.6 L m^−2^ h^−1^, while ionic rejection slightly improved. The use of starch reduced GO consumption by approximately 80%, providing a cost-effective solution for membrane enhancement. The hydrophilic nature of the starch–GO composite, its compatibility with the polypiperazinamide matrix and its ability to increase surface negative charge, combined with a thin top layer, contributed to the improved performance. Additionally, the membranes demonstrated excellent stability, which is attributed to the strong bonding between starch, GO and the polypiperazinamide layer. They also exhibited high rejection rates for both charged and neutral dyes, highlighting the potential of GO–starch composites in advancing polyamide membrane performance.

Dai et al. [94] reported that the distribution of GO and rGO depends on the solvent polar components, hydrogen content and polar surface energy. Materials with higher surface polarity (GO > rGO) exhibit greater stability in water and acetic acid solutions. Ma et al. [95] examined the influence of GO and rGO on starch nanocomposites, demonstrating that starch–GO composites exhibited superior properties compared to those with rGO. This improvement was attributed to GO’s higher concentration of polar agents and greater water solubility, which enhanced mechanical properties and dispersibility. The authors observed that incorporating 4 wt% GO into starch–GO nanocomposites resulted in an optimal level of water vapor permeability (WVP).

Water vapor readily permeated the plasticized starch (PS) film, which exhibited the highest WVP value of 5.68 × 10⁻^10^ g m^−1^ s^−1^ Pa^−1^. Incorporating 4 wt% of GO significantly reduced the WVP of GO/PS composites to 3.2 × 10^−10^ g m^−1^ s^−1^ Pa^−1^, whereas rGO/PS composites required a higher 8 wt% rGO loading to achieve a more modest decrease to 3.7 × 10^−10^ g m^−1^ s^−1^ Pa^−1^.

The effective dispersion of GO and rGO fillers within the PS matrix likely introduced tortuous diffusion pathways, impeding water molecule movement. However, GO established stronger interfacial interactions with the PS matrix than rGO, further restricting water passage in GO/PS composites.

Overall, these composites demonstrated moisture barrier properties comparable to pure PS films. Notably, GO/PS composites with lower GO loading exhibited superior moisture resistance compared to rGO/PS composites, which required higher rGO concentrations to achieve similar effects.

Starch–GO composites generally outperform starch–rGO composites due to the higher polarity of GO, which enhances mechanical properties and compatibility with starch. GO contains more oxygen functional groups than rGO, leading to better dispersion, stronger hydrogen bonding and improved structural integrity in starch-based nanocomposites.

Additionally, starch–GO composites exhibit higher hydrophilicity, making them more suitable for biomedical, packaging and environmental applications compared to starch–rGO composites, which tend to have lower solubility and weaker interfacial interactions [96].

Zheng et al. [97] studied the influence of plasticizers on starch–graphene nanocomposites using glycerol as a plasticizer for starch and starch-grafted graphene (GN–starch) as a nanofiller to produce PS–graphene nanocomposites. In this study, GO was reduced by hydrazine in the presence of starch, forming GN–starch. Starch acted as a stabilizing agent, preventing graphene restacking during the reduction process. Transmission electron microscopy (TEM) revealed that starch nanocomposites containing GO had a smoother and clearer surface compared to those with graphene. Upon oxidation, graphite sheets became flat and transparent with a wrinkled structure, whereas the presence of starch in GN–starch resulted in a darker appearance. Fourier transform infrared spectroscopy (FTIR) analysis showed that starch–graphene composites exhibited three characteristic peaks corresponding to C-O-H (1156 cm^−1^) and C-O-C (1080 and 980 cm^−1^) bonds. In contrast, GO displayed peaks associated with disordered regions (1350 cm^−1^) and graphene regions (1590 cm^−1^) [98]. Additionally, the impact of GO on glycerol-plasticized pea starch was investigated using a solution-casting method with water as the solvent with various GO concentrations (0, 0.4, 0.8, 1.0, 1.5, and 2.0 wt%). The morphological analysis of the bio-nanocomposite films revealed a shift in the characteristic FTIR peaks of starch to lower wavenumbers as GO content increased. In the neat starch film, the stretching and bending vibrations of the −OH groups were observed at 3283 cm^−1^ and 1650 cm^−1^, respectively. The bands at 1151 cm^−1^ and 1077 cm^−1^ corresponded to the stretching vibration of C–O in C–O–H groups, while the band at 997 cm^−1^ was associated with the stretching vibration of C–O in C–O–C groups. Additionally, the peak at 2928 cm^−1^ was characteristic of C–H stretching linked to ring methane hydrogen atoms. The C–O–C ring vibration peak in starch appeared at 765 cm^−1^. Upon the formation of PS/GO-5 biocomposite films, the characteristic peaks of PS at 1650, 1151, 1077 and 997 cm^−1^ shifted to 1642, 1145, 1072 and 993 cm^−1^, respectively. This shift suggests the establishment of hydrogen bonding interactions between GO and PS within the biocomposite films. In alignment with these findings, X-ray diffraction (XRD) results demonstrated a decline in starch diffraction peak intensity [99]. The neat starch film exhibited a typical C-type crystalline pattern, incorporating both A-type and B-type polymorphs, similarly to other legume starches. This structure was characterized by five primary diffraction peaks at 5.7° (B-type polymorphs), 15.1° (A-type polymorphs), 17.1° (common to both A and B types), 19.9° and 22.1° (B-type polymorphs). Following the incorporation of GO, the characteristic diffraction peak of graphite oxide was no longer observed, while the diffraction intensity of the PS film decreased. This suggests that GO sheets were effectively exfoliated and uniformly distributed within the PS matrix, influencing its crystalline structure. The observed changes may be attributed to the gelation process, during which the small-sized GO sheets penetrated starch polymer chains, forming hydrogen bonds with PS. This interaction likely restricted polymer chain mobility, leading to a significant reduction in the rate of PS re-crystallization.

Pregrino et al. [98] immobilized GO using potato starch (ST) to study the photochemical reduction and gas detection capabilities of GO films.

ST/GO films were built layer by layer (LbL) on quartz substrates through hydrogen bonding. Once assembled, the films underwent a UV-induced photochemical reduction (254 nm) of GO to rGO, following first-order reaction kinetics. The reduction process was notably more efficient when GO was paired with ST rather than with the non-oxygenated polyelectrolyte poly(diallyl dimethylammonium) chloride (PDAC). To evaluate gas-sensing performance, ST/rGO and PDAC/rGO sensors were fabricated via the LbL technique on gold-interdigitated microelectrodes. These sensors were tested under varying humidity conditions and in the presence of different concentrations of ammonia, ethano and acetone. The results indicated that sensors containing ST exhibit significantly higher sensitivity than those incorporating PDAC, particularly in humid environments. Additionally, an array of these sensors generated unique electrical responses for each analyte, allowing for precise identification and quantification across a broad concentration range (10–1000 ppm).

*Chitosan*, a renewable polymeric material, has attracted considerable attention due to its advantageous properties, including bacteriostatic activity, biodegradability, biocompatibility and biosensor suitability. It also regulates material leaching and release, is non-toxic, aids in lesion healing and is widely used in food packaging and texture enhancement [13,100]. However, its mechanical strength is a limiting factor. To improve this aspect, graphene has been integrated into chitosan-based composites, significantly enhancing their mechanical, thermal and barrier properties [101]. Wang et al. [102] synthesized a chitosan–rGO nanocomposite and examined its XRD profile, which confirmed that rGO did not alter chitosan’s crystalline structure. The inclusion of 6 wt.% rGO resulted in notable improvements in Young’s modulus, tensile strength, elongation at break and conductivity. Conversely, aggregation occurred at a higher rGO concentration (7 wt.%), leading to reduced conductivity [103]. In another study, Cheng et al. [104] demonstrated that rGO enhanced the adsorption efficiency and dye affinity of rGO–chitosan nanocomposites. Han et al. synthesized chitosan/GO composite films by mixing an aqueous solution of chitosan and GO in the presence of diluted acetic acid [105]. The structure, thermal stability and mechanical properties of the films were analyzed using wide-angle X-ray diffraction (WAXD), FTIR, scanning electron microscopy (SEM), AFM, thermogravimetric analysis (TGA) and mechanical testing. The experimental results confirmed that chitosan and GO blend homogeneously, leading to significant improvements in mechanical strength compared to pure chitosan films, particularly in their wet state. The tensile strength of the chitosan/GO (5:1, *w*/*w*) composite film was 1.7 times higher than that of pure chitosan in the dry state and 3 times higher in the wet state. Additionally, the storage modulus of the composite film remained high up to 200 °C, indicating enhanced thermal stability. These composite films exhibited great potential for applications in biomaterials and packaging, owing to their superior mechanical properties and stability.

*Cellulose*, a versatile biopolymer, is widely utilized in bioabsorption, cleaning and purification processes, as well as in the production of eco-friendly packaging and paper products [106]. Despite its many advantages, cellulose, like other biopolymers, has limited thermomechanical stability. To address this weakness, researchers suggest reinforcing it with nanofillers such as graphene or GO. Composed of glucose units linked by β-glycosidic bonds in parallel chains, cellulose differs from starch, which is water-soluble. Instead, it dissolves in specific solvents like N-methyl morpholine-N-oxide and N,N-dimethylacetamide lithium chloride. Notably, N-methyl morpholine-N-oxide enhances the interaction between cellulose and GO by forming hydrogen bonds, promoting better dispersion and exfoliation. This property makes it a sustainable solvent choice for cellulose processing [107]. The incorporation of GO into cellulose at concentrations of 0.2, 0.5, 1, 3 or 5 wt.% has been shown to enhance electro-thermal properties. Heating GO removes its oxygen-containing groups, thereby improving the electrical performance of the composite. Additionally, the formation of hydrogen bonds between cellulose and graphene contributes to increased mechanical strength and viscosity [108]. Research by Wu et al. [109] found that GO, when introduced into GO-LiOH-urea composites, significantly improved both tensile strength and elongation at break. Carboxymethyl cellulose (CMC), a highly soluble derivative of cellulose, has also been integrated into nanocomposites. For instance, Cheng et al. [110] developed a CMC-GO biosensing system using NaBH_4_ as a reducing agent and a correction electrode for hemoglobin immobilization. In this composite, CMC’s amphiphilic nature enhanced GO electron transfer ability, making it an effective material for biosensor applications.

*Alginates* have become a focal point in biomedical research due to their exceptional properties and diverse applications [111]. As with other biopolymers, alginate-based materials are non-toxic, biocompatible and biodegradable, making them suitable for food packaging and wound healing [112]. These biocomposites are capable of covering injured skin, absorbing exudates and aiding in the healing process [97]. Despite these advantages, alginates face challenges related to their mechanical properties and structural integration [113], which has led to efforts aimed at enhancing their performance. One approach is blending alginate with other polymers such as polyvinyl alcohol (PVA) and pectin [114], but these additives offer limited improvements. In contrast, incorporating inorganic materials has proven more effective in strengthening the alginate matrix.

Among these, carbon nanomaterials, particularly graphene and graphene GO, have shown promise in reinforcing alginate both mechanically and electrically. GO, with its high surface area and functional groups, facilitates strong interactions with polymeric matrices, promoting better dispersion and integration in composites [115]. The addition of GO to sodium alginate improves its mechanical properties, enhances biological functionality and enables controlled release [116,117]. For example, Ionita et al. [118] found that incorporating 6% wt. GO into an alginate nanocomposite increased Young’s modulus by approximately 300% and enhanced thermal stability. The FTIR analysis of pure sodium alginate revealed three key peaks (−OH, −COO and −C-O-C-), while the sodium alginate-GO composite exhibited an additional peak, indicating strong electrostatic interactions and hydrogen bonding between the two components, which contributed to improved thermomechanical properties [118].

*Lignin*, the second most prevalent natural polymer after cellulose, has recently attracted significant attention [119]. Lignin is a sustainable and cost-effective material rich in oxygen functional groups, making it ideal for modifying GO nanosheets [120]. Its biocompatibility and biodegradability also make it essential for developing bio-based products, a key aspect of comprehensive biorefinery concepts [121].

Lignin is a broad term encompassing various types of polymers, and its biocompatibility depends on its structure and processing method. Some lignins have shown promising biocompatibility, including lignosulfonates, which are water-soluble lignins derived from sulfite pulping and that are often used in biomedical applications thanks to their low toxicity and antioxidant properties [122]. Another one is Organosolv lignin, which is produced using organic solvents; this lignin type is known for its high purity and good compatibility with biological systems [123]. Also, modified lignin-based hydrogels have been explored for biomedical applications, including drug delivery and tissue engineering [124]. These lignins have been studied for their antibacterial, antioxidant and UV-protective properties, making them suitable for biomedical and pharmaceutical applications.

*Hyaluronic acid (HA)* is a naturally occurring polysaccharide originally sourced from bovine vitreous humour, rooster combs and *Streptococcus* bacteria [125]. Due to its advantageous properties—including biocompatibility, biodegradability, its viscoelastic nature and its derivation from renewable sources—HA has attracted attention as a biomaterial for various therapeutic uses [126]. Tao et al. demonstrated that paclitaxel could be released from HA-based hydrogels, attributing the release mechanism to repulsive interactions between ionized HA chains and the drug at elevated pH levels and increased swelling [127]. Crosslink density, pH and the formulation components influenced the extent of swelling and mesh expansion in the HA hydrogel. In a related study, Kaya et al. developed a drug-delivery matrix composed of electroactive polymeric films, integrating HA, gelatin, poly(ethylene oxide) and rGO. This system was used to investigate the release profile of irbesartan, which occurred over timeframes ranging from several hours to days depending on rGO concentrations [128]. Furthermore, HA has been widely employed as a drug-releasing matrix in several formulations—including hydrogels, multilayers, nanogels and microsphere-based systems—for the delivery of proteins, such as lysozyme [129], and antibiotics, like ciprofloxacin [130], levofloxacin [131] and rifampicin [132], as well as anticancer agents including paclitaxel and doxorubicin [133].

### 4.2. Protein-Based Graphene Bio-Nanocomposites

*Gelatin* is a natural, water-soluble protein obtained from partial or complete collagen hydrolysis. It appears as a white or yellowish, tasteless and odorless substance widely employed in medicine for tablets, capsule shells and powders [134]. Additionally, it serves as a matrix for cell culture and tissue engineering [135]. Due to its high protein content, gelatin can also substitute fat and carbohydrates [136]. Its abundant availability and biocompatibility make it highly suitable for food packaging applications. The triple-helical structure of gelatin provides significant physical strength, high water-binding capacity, film-forming ability and emulsification properties. These characteristics enable amino acids in gelatin to absorb UV radiation, thereby preventing oxidative damage in food products [137]. However, while gelatin films exhibit good mechanical and physical strength, they have limitations in temperature and drying tolerance. Under certain drying conditions, pure gelatin films can become rigid, inelastic and prone to breakage. They are also highly sensitive to moisture, which can cause swelling, dissolution or splitting. To address these limitations, incorporating nanomaterials can enhance their strength, thermal stability and moisture resistance [138,139]. GO and its nanocomposites have been shown to significantly improve gelatin’s mechanical and thermal properties. Wan et al. [140] developed a strong and biologically active GO–gelatin nanocomposite using a solution-casting method. The interaction between functional GO groups and gelatin polar amino acids, driven by hydrogen bonding, resulted in homogeneous and transparent nanocomposite films. The addition of 1% GO led to an 84% increase in tensile strength, a 65% rise in modulus and a 58% improvement in elongation at break. Similarly, Adilah et al. [141] prepared gelatin/GO composite films with GO concentrations ranging from 0.5% to 2.0% using a sonication method. Their findings indicated enhanced tensile strength, superior resistance to light and UV radiation, reduced film thickness and improved water resistance. Further advancements were made by Nassira et al. [142], who synthesized gelatin–GO nanocomposites by incorporating a natural antioxidant and reducing agent, ascorbic acid. They observed a rapid increase in electrical conductivity, with GO concentrations exceeding 0.5 vol %. Additionally, the composite exhibited a percolation threshold that was significantly lower than previously reported graphene-based nanocomposites and ceramic–polymer composites. The out-of-plane modulus of nanocomposite films containing 10% graphene nanosheets increased from 0.5 ± 0.07 GPa to 3.0 ± 0.7 GPa, highlighting a substantial improvement in mechanical strength. Hydrogels with double cross-linked network structures, where GO acts as a multifunctional cross-linker and reinforcing nanofiller, demonstrated enhanced mechanical properties, including greater stiffness and toughness [143]. The hydrogel rods, measuring 20 mm in height and 10 mm in diameter, were subjected to compression at a speed of 1 mm/min using a 50 N load cell. A novel approach involving Pickering emulsion was used to generate porous gelatin membranes, where GO stabilized the emulsion due to its amphoteric nature and π–π interactions with the EthB phase [134]. Various spectroscopic techniques, including FTIR, have confirmed strong interfacial interactions between gelatin and GO.

Differential scanning calorimetry (DSC) results indicate the good dispersion of GO nanosheets within a gelatin matrix, resulting in a large interfacial surface area. These interactions enhance stress transfer, contributing to higher tensile strength. As GO concentration increases, Tg values also rise, suggesting restricted gelatin chain mobility due to strong bonding between GO sheets and gelatin polar groups [133]. rGO, when modified with polar functional groups such as chitosan, exhibits similar reinforcing effects in hydrophilic polymers like gelatin. The combination of rGO and chitosan enhances mechanical strength in gelatin films, demonstrating a synergistic effect. Increasing rGO content leads to greater stiffness, as reflected in the decreased elongation at break [144]. While extensive research has been conducted on GO-incorporated gelatin polymer nanocomposites to enhance physicochemical, mechanical, thermal, morphological and biological properties for applications such as drug delivery, cytotoxicity and antimicrobial activity, studies specifically examining gelatin interactions with graphene-based nanomaterials (GBN) remain limited. Piao et al. [134] reported that incorporating graphene and GO nanosheets into gelatin-based nanocomposites significantly improves their thermomechanical properties. However, GO functionalization is essential to achieving the desired mechanical strength and ductility in gelatin–GO composites [145,146].

*Bovine serum albumin (BSA)* is a predominant serum protein, constituting nearly 60% of plasma proteins. It appears as a water-soluble white powder and remains stable within a pH range of 4.0–9.0 [147]. At room temperature, BSA maintains an α-helical secondary structure and is widely used in targeted drug delivery. Moreover, it serves as a model protein for studying protein interactions with nanomaterials [148,149].

Several studies have focused on the interaction between BSA and nanomaterials like GO to assess their influence on BSA’s structural integrity and biological activity. Nan et al. [150] employed spectroscopic and microscopic methods to investigate the binding behavior of BSA with GO. Their findings revealed that fluorescence quenching occurs through an initial static quenching mechanism, followed by dynamic quenching. The negative enthalpy (ΔH), entropy (ΔS) and Gibbs free energy (ΔG) values suggested that the interaction is governed by van der Waals forces and hydrogen bonding. This process was found to be both exothermic and spontaneous. Additionally, BSA underwent structural alterations upon contact with GO, leading to modifications in the amino acid microenvironment. Further research indicated that GO at lower concentrations primarily induces fluorescence quenching via a static mechanism, while higher concentrations result in both static and dynamic quenching. Shifts in fluorescence wavelengths signified GO-induced changes in the BSA microenvironment, accompanied by conformational rearrangements. Circular dichroism (CD) spectra supported these observations, showing a decline in BSA α-helical content [151]. The adsorption of BSA onto GO follows a pseudo-second-order kinetic model. The formation of a ground-state complex between BSA and GO leads to fluorescence quenching and modifications in both secondary and tertiary structures. This interaction results in decreased thermal stability and drug-binding affinity yet enhances esterase-like activity and non-enzymatic glycosylation [152]. Hydrogen bonding, hydrophobic interactions and π–π stacking—arising from BSA hydrophilic and aromatic residues—were identified as the primary driving forces for BSA-GO binding. Specifically, fluorescence quenching is predominantly attributed to hydrophobic and π–π interactions. The Rydberg constant (Rh) for fully denatured BSA was measured at 83.33 Å, whereas upon interaction with 70 µg/mL GO, the Rh value reduced to 58.2 Å, suggesting partial unfolding rather than complete denaturation. The α-helical content of BSA gradually declined with increasing GO concentrations, dropping from 55.6% to 50.2% at 5 µg/mL GO, 45.7% at 15 µg/mL GO, and 41.3% at 75.5 µg/mL GO. These findings confirm that BSA undergoes conformational adjustments upon adsorption onto GO but retains some of its native structure [153].

Bapli et al. [154] analyzed the interaction between BSA and 7-(Diethylamino)coumarin-3-carboxylic acid (7-DCCA) in the presence and absence of GO through spectroscopic, calorimetric and microscopic techniques. Their study reported an increase in BSA absorbance with a blue shift upon 7-DCCA addition, evidencing complex formation with or without GO. The fluorescence intensity of the BSA-7-DCCA complex diminished more significantly when GO was present, accompanied by a blue shift, which was attributed to the relocation of Trp residues into a solvent-free microenvironment. Additionally, the fluorescence intensity decrease in the absence of GO was linked to alterations in the ionization state of the BSA ε-amino group. Despite strong binding between BSA and GO, the energy transfer between BSA and 7-DCCA remained unchanged.

The adsorption of BSA onto amine-functionalized GO (NGO) is influenced by the system’s pH. In acidic conditions, BSA exhibits a stronger binding affinity compared to neutral or basic environments. At pH 7.4, its adsorption onto NGO decreases due to its compact structure and electrostatic repulsion from negatively charged NGO groups. In alkaline conditions, electrostatic repulsion between negatively charged NGO and BSA further limits binding. The fluorescence quenching mechanism observed in these interactions involved a combination of static and dynamic quenching, with greater quenching effects in acidic media [155].

*Amylase* is an enzyme responsible for breaking down starch and glycogen into simpler sugars like maltose and glucose by cleaving the glycosidic bonds between glucose units in the polysaccharide chains. Different amylases have distinct optimal pH and temperature ranges for their catalytic action. For example, salivary amylase works best in slightly acidic to neutral conditions, whereas pancreatic amylase is most effective in a slightly alkaline environment. Besides humans and animals, some plants, like those with germinating seeds, also produce amylase to convert stored starch into glucose, providing energy during germination. Liu et al. [135,156] explored the influence of GO on the structure and biological function of α-amylase. This study revealed that GO forms a ground-state complex with amylase through weak interaction forces. This interaction caused a reduction in the intrinsic fluorescence intensity of amylase via a static quenching mechanism. Additionally, the presence of GO altered the fluorophore’s microenvironment and impacted the enzyme’s secondary structure. Importantly, this interaction also led to a decrease in amylase catalytic activity.

*Wheat gluten* is a widely used biopolymer due to its low cost and ability to form composites with excellent mechanical properties and effective oxygen barrier capabilities. However, its primary limitation is its poor resistance to water and moisture [157,158]. To address this issue, chitosan can be combined with wheat gluten, resulting in a composite with enhanced mechanical strength and better moisture resistance [159,160]. Despite these improvements, the properties of wheat gluten-based composites still fall short, and nanofillers have emerged as the most effective solution for further enhancements [161]. A wide range of nanomaterials can be utilized to improve the properties of these composites [162,163]. Graphene nanofillers, in particular, hold significant promise when paired with biopolymers like gluten due to their excellent thermomechanical and electrical properties [164]. However, one of the main challenges in using graphene with gluten is the tendency for graphene nanoplates to agglomerate. To overcome this, GO should be used to improve dispersibility and uniform distribution in the polymer matrix [165]. Research indicates that wheat gluten–GO composites offer superior thermo-mechanical properties compared to wheat gluten–graphene composites [166]. These enhanced properties make wheat gluten–GO composites suitable for a variety of applications, including food packaging, drug delivery, gene therapy and cleaning [167].

*Soy proteins*, derived from soybeans, are among the most essential biopolymers. These proteins are cost-effective, durable, biodegradable and environmentally friendly [168]. Soy protein consists mainly of two components, glycinin and β-conglycinin, which are rich in amino acids like aspartic acid, lysine, phenylalanine, leucine, glutamic acid and tyrosine. Song et al. [169] explored the versatile uses of soy protein, emphasizing its outstanding potential in creating sustainable food packaging materials. Additionally, soy protein serves as an effective chelating agent for metal ions, which makes it valuable in developing biosensors for removing heavy metals from wastewater [170]. Despite its advantages, soy protein faces limitations, such as poor mechanical properties and high solubility in acidic environments, which restrict its broader applications [162]. To enhance its thermomechanical properties, soy protein has been combined with various substances, including synthetic polymers, inorganic/organic materials and biological or medicinal compounds [171]. In 2010, Lee et al. [172] developed a soy protein composite with montmorillonite, investigating its potential in drug delivery systems. Further studies by Zhuang et al. [173] focused on a soy protein–GO nanocomposite, which demonstrated effective functionality in removing antibiotics from different solutions. The research highlighted that the soy protein–GO nanocomposites exhibited better hydrophilicity, biological activity and lower toxicity compared to pure graphene. The composite contains numerous sulfhydryl groups and tyrosine amino acids, which act as reducing agents, reducing GO to produce rGO. The amphiphilic structure of soy protein allows it to adhere to the polymer matrix, enhancing the composite’s ability to bond with solid surfaces. This characteristic enables soy protein to reduce GO and form a stable link with the rGO surface, creating a hybrid nanocomposite [126].

### 4.3. Other Biodegradable Polymers in Graphene-/GO-Based Bio-Nanocomposites

Various biopolymers can be conjugated to GO; among them, polyethylene glycol (PEG) and polylactic acid (PLA) offer highly desirable properties. PEG and PLA conjugated with GO provide enhanced mechanical strength, thermal stability and biocompatibility compared to traditional biodegradable polymers such as PBS, PBAT, PCL, PHA and PHB. The incorporation of GO enables improved surface functionalization and tunable degradation rates, making PEG/PLA-GO composites highly versatile for biomedical and advanced applications. Unlike PBS and PBAT, which often have slower or less controllable degradation, and PCL, PHA and PHB, which may lack mechanical robustness or functional versatility, PEG and PLA with GO create a multifunctional platform that combines biodegradability, controlled drug release and potential electrical conductivity, thus expanding their applicability beyond standard biodegradable polymers.

*PLA* is a sustainable and biodegradable polymer with diverse applications, including packaging materials and medical applications such as tissue engineering and wound healing. Researchers have enhanced PLA by incorporating graphene nanofillers into its matrix, forming nanocomposites that demonstrate superior thermomechanical behavior and improved crystallinity compared to unmodified PLA [174,175]. Solvents such as N, N-Dimethylformamide (DMF), acetone and chloroform are commonly used to disperse graphene within the PLA matrix. The selection of an appropriate solvent is influenced by the Hansen solubility parameter (HSP), which governs polymer–solvent interactions. Among the solvents tested, DMF provided the most efficient dispersion of graphene, followed by acetone, with chloroform being the least effective in this context [176]. A study by Cao et al. [167] explored how the solubility of different forms of graphene—specifically lyophilized versus vacuum-filtered—varies in DMF when integrated into PLA. The results showed that lyophilized graphene dispersed more uniformly, which contributed to enhanced thermomechanical properties in the resulting nanocomposites. SEM observations revealed a strong interface between PLA and graphene sheets. Meanwhile, AFM analysis indicated that lyophilized graphene exhibited a reduced thickness compared to single-layer GO, likely due to the elimination of oxide groups during processing. XRD patterns revealed minimal differences between pure PLA and PLA containing 2% graphene by weight, suggesting that graphene was dispersed as individual layers within the matrix. The tortuous network formed by graphene impeded the release of volatile substances. DMF proved to be an especially effective solvent in facilitating this dispersion [133]. However, increasing graphene or GO concentrations in PLA composites were associated with a decreased crystallization rate [177]. Pinto et al. [178] investigated the influence of plasticizers on PLA–graphene and PLA-GO composites. Their findings indicated that plasticizer-modified films displayed greater mechanical strength, including improved tensile strength and Young’s modulus, compared to PLA composites without plasticizers. Additionally, the presence of graphene or GO resulted in an elevated glass transition temperature (Tg). The Tg for PLA–graphene composites was notably higher than for PLA-GO, a difference attributed to the tendency of GO sheets to aggregate due to their flexible chain structure. This issue can be addressed by incorporating GO through in situ melt polycondensation, which minimizes agglomeration [179,180]. Pandey and co-workers [181] fabricated GO using pencil lead as a carbon source through a modified version of Hummers’ method. The resulting GO was then incorporated into PLA to produce composite materials, with GO concentrations ranging from 0.25 to 1.25 wt%. The structural and chemical properties of the synthesized GO were characterized using a variety of techniques, including XRD, energy-dispersive X-ray spectroscopy (EDX), Raman spectroscopy, and FTIR. Test samples were produced via a syringe-based direct ink writing process to evaluate both mechanical and electrical performance. Among all composites, the highest tensile strength was recorded at 0.5 wt% GO concentration, while Young’s modulus peaked at 1.25 wt% GO. Moreover, the samples with 1.25 wt% GO exhibited superior compressive strength and microhardness compared to other formulations. In terms of electrical properties, the composite containing 1.25 wt% GO demonstrated the greatest electrical conductivity, reaching 58.91 µS/m. On the other hand, the maximum dielectric constant of 307.69 at 100 Hz was measured in a sample with 1 wt% GO. Overall, these results indicate that incorporating relatively low concentrations of GO significantly improved the mechanical and electrical characteristics of the PLA matrix.

*PEG* is a man-made polymer that is highly water-attracting and adaptable, available in various chain lengths and equipped with a wide range of functional end groups [182]. Its derivatives, which come in different molecular weights, find extensive use across a broad spectrum of applications [183]. PEG has received approval for inclusion in food products, cosmetic formulations and a variety of pharmaceutical uses [184]. It is also a common ingredient in beauty products, surface disinfectants for skin and many household formulations [101]. In topical products, PEG serves multiple functions, such as maintaining moisture, stabilizing mixtures, enhancing skin absorption and conditioning the skin [184]. By attaching PEG molecules to the surface of GO, it becomes possible to address several limitations without sacrificing GO’s beneficial features. PEG-functionalized GO exhibits improved compatibility with biological systems, reduces the chance of triggering immune responses, decreases unwanted interactions, increases solubility and resists clumping in bodily fluids. Furthermore, this modification extends the material’s circulation time in the bloodstream, helping it accumulate more efficiently at target locations and enhancing the therapeutic impact [185,186]. As a result, PEGylated GO stands out as an important innovation that significantly boosts GO’s effectiveness in biomedical contexts.

## 5. Biological Applications of Graphene-Based Bio-Nanocomposites

### 5.1. Drug Delivery Systems

One of the main challenges modern medicine is facing is the creation of novel and efficient drug delivery methods that can enhance therapeutic agents’ therapeutic profile and efficacy. Numerous novel medication delivery methods have been developed as a result of developments in nanoscience and nanotechnology that have allowed the synthesis of new nanomaterials [187]. Since graphene’s discovery, there has been a surge in studies using this novel material for drug delivery applications. Wang et al. [188] explored the role of GO as a drug carrier within konjac glucomannan (KGM)/sodium alginate (SA) bio-nanocomposite systems for anticancer therapy. The pursuit of novel carriers that enable the controlled release of anticancer agents remains an active area of research [189]. In the early stages of development, natural biopolymers such as starch, guar gum, chitosan, KGM and SA have been extensively investigated for this purpose [190,191]. These materials are valued for their sustainability, biodegradability, compatibility with biological systems and lack of toxicity. Nevertheless, biopolymers in their unmodified form often exhibit poor mechanical strength, and regulating drug release from them remains a significant challenge. This limitation arises from the inherently weak interactions between biopolymeric matrices and loaded drugs. Furthermore, biopolymers tend to degrade quickly during the drug delivery process, which hampers their performance. To overcome these issues, researchers have introduced nanomaterials like GO into the polymeric matrix to enhance functionality [192,193]. These GO-based bio-nanocomposites have garnered significant interest due to their improved capabilities in enabling precise control over drug release. GO, being a single-atom-thick sheet with oxygen-containing functional groups, exhibits a strong potential as an effective drug delivery platform [194]. Maturavongsadit et al. [195] developed a new method for the localized and prolonged release of Senexin A (SenA), a cyclin-dependent kinase 8/19 inhibitor and promising therapeutic agent for occlusive vascular disorders. The approach involved the use of HA-based hydrogels integrated with GO nanosheets to facilitate the encapsulation and delivery of SenA. GO nanosheets enabled non-covalent interactions with SenA, aiding its effective incorporation into the hydrogel matrix. The engineered hydrogels successfully enabled sustained SenA release for 21 days, with the added advantage of providing adjustable release profiles. Furthermore, in vitro experiments confirmed that the hydrogels exhibited biocompatibility, supporting their safe use in biomedical contexts. This innovative hydrogel system, combining GO and HA, presents a promising solution for perivascular drug delivery, specifically targeting conditions like vein graft failure associated with occlusive vascular disease. In another study, Sittisanguanphan [196] and co-workers developed drug delivery matrices composed of HA- and GO-enhanced HA hydrogels for the controlled release of tamoxifen citrate (TMX), an anticancer agent, under the influence of an applied electric field. The hydrogels, prepared using solution casting and citric acid as the crosslinking agent, served as transdermal patch platforms—where pristine HA functioned as the base matrix and GO acted as the encapsulation medium for TMX. To evaluate drug release performance, in vitro experiments were conducted using a modified Franz diffusion cell, focusing on the influence of crosslinking density, electrical stimulation and GO inclusion. The release kinetics of TMX were governed by three distinct transport mechanisms, classical Fickian diffusion, anomalous (non-Fickian) diffusion and Super Case II transport, depending on the extent of crosslinking. Lower crosslinking ratios in the pristine HA hydrogels led to increased TMX diffusion and overall release. When subjected to an electric field, the hydrogels exhibited enhanced drug diffusion and release, attributed to the electro-repulsive interactions between the charged matrix and the TMX molecules. Additionally, the presence of GO in the HA hydrogel significantly amplified TMX diffusion—by nearly two orders of magnitude—compared to HA hydrogels without GO, confirming GO’s effectiveness as a drug-hosting component.

Islam et al. [197] designed a nanodrug delivery platform incorporating podophyllotoxin (PTOX), a well-established anticancer compound, onto a GO base. Alongside drug delivery, the system’s effectiveness in inhibiting the enzymes α-amylase and α-glucosidase was also evaluated. PTOX was extracted from the roots of *Podophyllum hexandrum*, yielding 2.3%. GO was synthesized using Hummers’ method and subsequently functionalized into carboxylated GO (GO-COOH). This was then surface-modified with PEG in a 1:1 ratio within an aqueous solution to generate PEG-functionalized GO (GO-PEG). PTOX was then efficiently loaded onto GO-PEG, achieving a 25% drug loading rate. The resulting materials were thoroughly characterized through Fourier transform infrared (FTIR) spectroscopy, UV/visible spectroscopy and SEM. The FTIR analysis of GO-PEG-PTOX revealed a decrease in acidic functional groups and the emergence of ester bonds, indicating the successful attachment of PTOX to the GO framework. UV/Visible spectra showed enhanced absorbance in the 290–350 nm range for GO-PEG, further confirming effective drug loading (25%) onto the nanocarrier. SEM images depicted GO-PEG-PTOX as having a coarse, aggregated morphology with irregular distributions and visible PTOX binding on the surface. The GO-PEG-PTOX complex demonstrated strong inhibitory activity against both α-amylase and α-glucosidase, with IC_50_ values measured at 7 mg/mL and 5 mg/mL, respectively—values that are comparable to pure PTOX, which showed IC_50_ values of 5 mg/mL and 4.5 mg/mL. Notably, the nanocomposite achieved 25% drug loading and released 50% of the loaded PTOX over a 48 h period, highlighting the system’s therapeutic potential. Complementary molecular docking studies revealed four distinct interaction modes between PTOX and the active sites of the target enzymes, aligning well with the experimental findings. In summary, this work introduces a novel PTOX-loaded GO-based nanocomposite as a promising inhibitor of α-amylase and α-glucosidase in vitro, which was reported for the first time. In another investigation carried out by Jihad et al. [198], GO nanoparticles with a flaky morphology were synthesized via Hummers’ method and subsequently functionalized with PEG through an esterification process. These PEG-coated nanoparticles were then loaded with *Nigella sativa* seed extracts. The study aimed to assess their suitability as a smart drug delivery platform targeting *Staphylococcus aureus* and *Escherichia coli*. A thorough analysis of the spectral and structural properties of the GO-PEG nanoparticles was performed using techniques such as XRD, AFM, TEM, FTIR and UV/visible spectroscopy. Functionally, this nanodrug delivery system demonstrated antibacterial efficacy by disrupting bacterial structure. It was found to penetrate the nucleic acids and cytoplasmic membranes of the bacterial cells, ultimately compromising cell wall integrity, causing nucleic acid degradation and increasing membrane permeability. These findings highlight the potential of the GO–PEG–*N. sativa* formulation as a promising antibacterial agent.

### 5.2. Tissue Engineering

Natural healing can occur for bone injuries and defects that are smaller than a critical size. However, when the defect exceeds a certain size, typically larger than 800 μm to 1 mm, a template is necessary to support the repair process [199]. Tissue engineering has emerged as a promising solution for achieving long-term bone repair, with 3D scaffolds serving as effective templates for regeneration [200,201]. One approach to fabricating these scaffolds is through 3D printing, which is used to create nanocomposite scaffolds from PLA combined with hardystonite (HT) and GO [202]. In this process, GO acts as a coupling agent that facilitates the integration of alkaline-treated HT nanoparticles within the PLA matrix. The addition of HT-GO nanoparticles up to 30 wt% to the PLA matrix led to a noticeable increase in the material’s degradability, enhancing it from 7.33 ± 0.66% to 16.03 ± 1.47% over 28 days. In particular, the incorporation of 20 wt% HT-GO nanoparticles into the PLA scaffold (referred to as PLA/20HTGO) resulted in a significant boost in mechanical strength. Compressive strength increased from 7.65 ± 0.86 MPa to 14.66 ± 1.01 MPa, and the elastic modulus rose from 94.46 ± 18.03 MPa to 189.15 ± 10.87 MPa. The nanocomposite scaffolds also exhibited excellent bioactivity, as evidenced by the formation of apatite on the scaffold surface when immersed in simulated body fluid for 28 days. Furthermore, the PLA/20HTGO scaffold effectively promoted the adhesion and growth of MG63 cells, as well as the osteogenic differentiation of rat bone marrow mesenchymal stem cells. The overall findings indicate that the optimized PLA/20HTGO nanocomposite scaffold holds significant promise for applications in hard tissue engineering.

Akbari and co-workers [203] developed a novel green strategy to reduce GO and concurrently decorate it with strontium nanoparticles (SrNPs). This was achieved without the use of toxic reducing agents like hydrazine or additional chemical linkers. Hydrazine is commonly used for GO reduction, but it poses significant safety and environmental risks that need to be considered.

Instead, BSA served a dual purpose: acting both as a reducing agent for GO and as a stabilizing agent for the formation and attachment of SrNPs on the rGO surface. The successful reduction of GO by BSA and the simultaneous decoration of SrNPs were verified through UV/visible spectroscopy, FTIR and Raman spectral analysis. Microscopic evaluation using FE-SEM and TEM confirmed that the strontium nanoparticles were formed in situ and displayed a consistent size range of approximately 25 to 30 nanometers. Cell viability tests using the MC3T3-E1 preosteoblast cell line showed that the SrNPs-rGO composite significantly enhanced cell proliferation compared to both GO and BSA-rGO when applied at identical concentrations. Moreover, an assessment of alkaline phosphatase (ALP) activity revealed that Sr-BSA-rGO nanosheets stimulated higher osteogenic activity than the other tested materials. Gene expression studies further demonstrated that treatment with Sr-BSA-rGO led to a marked increase in the expression of osteogenesis-related markers, specifically RUNX2 and collagen type I (Col1). This research illustrates the effectiveness of using biocompatible molecules such as proteins for the eco-friendly decoration of functional nanoparticles on nanomaterials. Such methods offer a sustainable and non-toxic alternative to conventional chemical reduction processes involving harmful substances like hydrazine. Collectively, these findings indicate that Sr-BSA-rGO exhibits excellent potential to support bone tissue regeneration and may serve as a valuable osteoinductive material in orthopedic implants.

Significant research efforts have focused on advancing PEG-GO as an effective delivery vehicle for hydrophobic drug compounds. In contrast, its exploration within the field of tissue engineering remains relatively limited. Table 3 briefly highlights the role and potential applications of PEG-GO in the development of tissue scaffolding systems.

### 5.3. Antimicrobial Activity

Food microbial spoilage is a major contributor to food waste, which in turn leads to increased environmental pollution [209]. Yang et al. [210] highlighted that mold contamination affects at least 25% of fruits, posing a threat to human health and food safety. *Aspergillus niger* and *Bacillus subtilis* are among the most common food-contaminating microorganisms. Currently, the majority of food packaging materials are derived from synthetic polymers, which not only lack antimicrobial properties but are also non-biodegradable, contributing to environmental degradation [211]. As a result, one of the key objectives in the food industry is to develop biodegradable packaging materials with effective antimicrobial properties. In this regard, bio-nanocomposites have emerged as promising alternatives, setting a new direction for food packaging innovation [212].

In their study, Joz Majidi et al. [213] examined the antibacterial performance of GO-chitosan bio-nanocomposites against both Gram-positive (*S. aureus*) and Gram-negative (*E. coli*) bacteria. Their results showed that bacterial growth was significantly inhibited after 12 h of exposure to the bio-nanocomposites. The synergy between GO and chitosan enhanced the antimicrobial activity, with effectiveness increasing over time. This is largely attributed to the interaction between GO functional groups and the amine groups in chitosan, facilitating better adsorption and microbial interaction. The simultaneous presence of GO and chitosan also promotes the generation of reactive oxygen species, thereby amplifying the bactericidal properties of the material. According to the study, *S. aureus* was more susceptible to GO-chitosan bio-nanocomposites than *E. coli*, which is likely due to differences in bacterial cell wall structures and the enhanced resistance of *E. coli* membranes to the sharp edges of GO sheets [214]. The study also noted that *S. aureus* growth could be effectively suppressed for over 8 h using these composites. Further work by Xu et al. [215] involved the fabrication of GO–chitosan bio-nanocomposites and the evaluation of their antimicrobial capabilities. GO is known for its outstanding physicochemical attributes, including large surface area, superior thermal conductivity, high mechanical strength and its excellent potential as a carrier material [216]. Akhavan and Ghaderi [217] emphasized that the inclusion of GO in chitosan matrices enhances antimicrobial effects and boosts the composite’s mechanical integrity. The mechanism involves graphene nanoplatelets damaging bacterial membranes through their sharp edges, thereby compromising the cell wall’s integrity and leading to the leakage of intracellular contents [218]. In their study, Grande et al. [218] reported that GO–chitosan composites demonstrated superior antimicrobial and physicomechanical properties compared to chitosan alone, primarily due to strong crosslinking interactions between GO and chitosan. Their findings revealed that a bio-nanocomposite containing 0.6 wt% GO significantly inactivated *E. coli* and *B. subtilis* by 22.83% and 54.93%, respectively. Krystyjan and co-workers [219] investigated the functional characteristics of innovative bio-nanocomposites composed of a starch/chitosan matrix infused with GO nanoparticles, synthesized through an environmentally friendly approach. To evaluate their barrier efficiency, water vapor transmission rates were measured in both thin (0.095–0.107 mm) and thick (0.205–0.219 mm) film variants. The thinner composites demonstrated superior barrier performance and were therefore chosen for more in-depth examination. The films showed a notable susceptibility to degradation when exposed to enzymatic and acidic environments, highlighting their considerable biodegradability. Cytotoxicity evaluations revealed that the presence of GO did not adversely affect cell viability, nor did it significantly compromise DNA integrity in blood-derived cells, confirming the non-toxic nature of the materials. The composites also proved effective in limiting microbial proliferation, particularly against strains such as *Enterobacteriaceae*, *Escherichia coli* and *Campylobacter spp.*, suggesting strong antimicrobial potential. Both chitosan and GO were found to contribute to the inhibition of Gram-negative and Gram-positive bacteria, supporting their bactericidal properties. In addition to these qualities, the materials exhibited excellent optical transparency, which enhances their value as components of active packaging systems. Given these combined properties—biodegradability, safety, microbial resistance and optical clarity—the developed bio-nanocomposites show strong promise for practical use in the food packaging sector.

### 5.4. Wound-Healing Applications

The skin plays a critical role in defending the body against microbial infections and shielding it from external physicochemical harm. In fact, one of the key objectives in tissue engineering is to develop skin substitutes that offer effective protection while maintaining high biocompatibility, controlled biodegradability and favorable mechanical strength [220]. Synthetic polymers are known for their robust mechanical performance and typically hydrophobic characteristics, but they often lack the necessary biological recognition sites to support cellular activities. On the other hand, natural biopolymers exhibit excellent compatibility with biological tissues and degrade readily in physiological environments, though their mechanical strength tends to be insufficient [221]. As a result, materials that integrate the advantages of both synthetic and natural polymers have shown great promise in advancing the field of skin tissue engineering [222].

Among biopolymers, chitosan stands out as an effective option for wound-healing purposes [223]. It possesses excellent biocompatibility, high biodegradability and inherent antibacterial activity, making it a strong candidate for skin repair applications. For example, Yang et al. [224] developed chitosan/polyvinyl alcohol/GO (CS/PVA/GO) nanofibers incorporating antibiotic drugs like ciprofloxacin and ciprofloxacin hydrochloride using an electrospinning method. The drug release profile demonstrated a controlled release without an initial burst, with the addition of GO enhancing the drug release rate. The antibiotic-loaded nanofibers exhibited strong antibacterial properties against both Gram-negative and Gram-positive bacteria, as well as cytocompatibility with melanoma cells. In a different study, an increase in GO content within the electrospun CS/PVA/GO nanofibers led to a reduction in the thermal stability of the composite nanofibers [225]. The nanofiber web displayed significant antibacterial activity against both Gram-negative and Gram-positive bacteria, indicating its potential as an effective wound-dressing material.

GO, as highlighted by Goenka et al. [226] and Terzopoulou et al. [227], exhibits a unique combination of properties—including a large surface area, amphiphilicity, chemical resilience, strong mechanical features and notable antimicrobial effectiveness—positioning it as a valuable additive in biomedical composites [228]. Several studies have successfully integrated GO into various polymeric matrices to improve their functionality. For instance, Ionita et al. [229] developed a GO–gelatin–polyvinyl alcohol (PVA) composite, while Mahmoudi et al. [230] and Ye et al. [231] fabricated GO–chitosan and GO–cellulose composites, respectively. These composites demonstrated enhanced hydrophilicity, thermal and mechanical stability, bacteriostatic behavior and wound-healing capabilities—attributable to the strong interfacial bonding between GO and the host polymer. The incorporation of GO into polymer matrices has the potential to advance tissue engineering and wound-healing technologies significantly. GO surface functional groups play a key role in forming strong interactions with the surrounding polymer network, which contributes to the material’s enhanced performance. Lu et al. [232] reported that wound healing using graphene- or GO-based composites surpassed that of unmodified polymeric materials. Furthermore, the superior antimicrobial properties observed in these composites were associated with the disruptive impact of GO or graphene on microbial cell structures.

### 5.5. Food Industry Applications

The food industry, valued at several trillions of dollars globally [233], stands among the largest sectors and continues to progress steadily by integrating cutting-edge technologies and exploring innovative nanomaterials such as graphene. Desai et al. [234] described the successful immobilization of a commercially available amylase onto GO-Fe_3_O_4_ nanoparticle surfaces. The binding between amylase and the GO-Fe_3_O_4_ support was verified using XRD, FTIR and TGA. SEM and TEM provided additional structural evidence of the immobilization. Experimental parameters for the process were fine-tuned using the Plackett–Burman design, along with central composite design (CCD) approaches. Thermodynamic and biochemical evaluations revealed the improved stability of enzymes under higher temperature and pH conditions following immobilization. As predicted, the enzyme exhibited an increased Km value and a reduced Vmax, indicating altered catalytic efficiency post-binding. The operational performance of the immobilized enzyme is closely aligned with previously documented outcomes. Furthermore, the system demonstrated effective utility in producing syrup with a high maltose content, pointing to its commercial potential.

The sugar industry plays a vital role within the broader food and agriculture sector, significantly supporting the economies of various nations [235]. Both the upstream and downstream stages of sugar production, along with the manufacture of alternative sweeteners (ASTs), generate a range of colored and colorless byproducts—collectively referred to as sugar-derived contaminants (SDCs)—which can negatively impact product quality, ecosystems and human health [236]. To address this issue, a sustainable aerogel (PSPI/PGO) was developed by Xiong et al. using amine-modified soy protein isolate (SPI) and GO, designed specifically for the effective adsorption of multiple sugar derivative contaminants [237] (SDCs). The material demonstrated exceptional equilibrium adsorption capacities of 1124.2 mg/g for melanoidins, 291.1 mg/g for caramels and 399.3 mg/g for saccharin, outperforming previously reported adsorbents. Comprehensive characterization confirmed that PSPI/PGO possesses a porous structure with notable hydrophilicity, thermal resilience, non-toxic properties and long-lasting adsorption performance. Advanced kinetic models—such as AOAS, EXT-INT and LF-PC-AS—alongside quantum chemical calculations (including ESPL, ALIE, FMO, IGMH and HSA), indicated that charge interactions are the primary mechanism for SDC binding, with hydrogen bonding and other intermolecular forces playing secondary roles. In many of these processes, PSPI/PGO functioned as a hydrogen bond acceptor. The aerogel efficiency was also demonstrated in real wastewater treatment scenarios. Its biodegradability and suitability for further environmental applications were confirmed through lifecycle assessments, including soil burial tests, wastewater purification and garlic hydroponic growth trials using exhausted PSPI/PGO, highlighting its industrial promise and ecological compatibility.

Packaging materials play a crucial role in everyday life. When it comes to food, they are indispensable for maintaining the freshness and safety of products throughout their intended shelf life. Additionally, they contribute significantly to the efficient use of space during transport, storage and handling, thereby helping to reduce overall waste [238]. Wheat-derived materials are remarkably adaptable and widely employed in food packaging applications, ranging from edible surface coatings to durable structural film production. In a related investigation, Wu et al. [239] created films infused with rGO using vacuum filtration techniques. The incorporation of rGO notably improved the mechanical strength of the films, with an 8% rGO concentration resulting in a striking 639.8% increase in tensile strength, reaching up to 88.70 MPa. Furthermore, the presence of rGO decreased the film’s hydrophilicity, water vapor transmission and swelling capacity. For instance, the contact angle of the 10% rGO-based film was twice that of the control film (0% rGO), signifying enhanced hydrophobic characteristics. Water vapor permeability dropped by 10.7% as well. In another study, Ahmed et al. [240] employed calcium ions (Ca^2^⁺) to cross-link lignocellulosic fibers obtained from wheat straw. Elevating CaCl_2_ concentrations to 800 mM significantly reduced the film’s moisture content, solubility in water, vapor permeability, transparency and elongation capacity. Under these conditions, the films achieved a tensile strength of 6.61 ± 0.07 MPa—2.5 times greater than that of conventional polyethylene films—demonstrating the considerable mechanical reinforcement achieved through ionic cross-linking.

## 6. Conclusions

Graphene and its derivatives possess exceptional properties suited for diverse applications, whether used alone or within polymer matrices. Producing graphene or GO composites requires the careful selection of production methods to ensure efficiency and cost-effectiveness. Exfoliation-based GO synthesis enhances nanocomposites’ thermal and mechanical performance, even at low concentrations. Generally, graphene and GO add durability and strength to nanocomposite structures. In biopolymer systems, they improve conductivity, structural integrity and barrier properties due to strong interactions with hydroxyl, carboxyl and amino groups. Graphene also disperses more effectively than many other nanofillers. Bio-nanocomposites incorporating graphene support drug delivery, antimicrobial treatments and tissue engineering. Researchers further enhance graphene’s functionality through surface modifications and encapsulation.

With rising demand, the global graphene and GO trade is expected to grow by 60% annually. Extensive research into graphene–polymer and graphene–nanoparticle combinations continues to drive medical and biotech advancements. Functionalized graphene, GO and rGO demonstrate significant effectiveness in drug delivery, tissue engineering, bioimaging, MRI and biosensing. Oxygen-rich functional groups on graphene improve biosensor performance by enhancing signal response, surface area and biomolecule detection.

## Figures and Tables

**Table 1:** Main graphene and GO synthetic methods.

		Method	Process Used	Advantages	Disadvantages
Top-Down	Oxidation (GO synthesis)	Brodie’s Method	KClO_3_, HNO_3_	High Oxidation Efficiency: Produces highly oxidized GO with a well-defined structure. Controlled Functionalization: Allows for precise tuning of oxygen-containing functional groups. Historical Significance: One of the foundational methods for GO synthesis, providing insights into oxidation mechanisms.	Harsh Reaction Conditions: Requires strong oxidizers (KClO_3_ and HNO_3_), which pose safety risks. Environmental Concerns: Generates hazardous waste, including nitrogen oxides (NOx), which contribute to pollution. Lower Yield: Compared to modern methods like Hummers’ Method, Brodie’s approach is less efficient in terms of GO production. Time-Consuming: The reaction process is slow, requiring multiple oxidation steps.
		Staudenmaier’s Method	KClO_3_, H_2_SO_4_, HNO_3_	Higher Oxidation Efficiency: Compared to Brodie’s method, Staudenmaier’s approach achieves greater oxidation, leading to better exfoliation of GO. Improved Yield: The addition of sulfuric acid enhances the oxidation process, resulting in higher GO production. Controlled Functionalization: Produces oxygen-rich GO, which can be useful for surface modifications and chemical applications.	Harsh Reaction Conditions: Requires strong acids and oxidizers, posing safety risks during handling. Environmental Concerns: Generates hazardous waste, including nitrogen oxides (NOx), which contribute to pollution. Lower Electrical Conductivity: Due to high oxygen content, GO produced by this method may require additional reduction steps to restore conductivity.
		Hummers’ Method	KMnO_4_, H_2_SO_4_, NaNO_3_	Efficient Oxidation: Produces highly oxidized GO with a well-defined structure. Scalability: Suitable for bulk production, making it viable for industrial applications. Safer Than Older Methods: Developed as an improvement over Staudenmaier’s method, reducing explosive risks. Controlled Functionalization: Allows for precise tuning of oxygen-containing functional groups.	Environmental Concerns: Generates hazardous waste, including nitrogen oxides (NOx), which contribute to pollution. Strong Acid Usage: Requires high concentrations of H_2_SO_4_, posing corrosive and handling risks. Residual Functional Groups: It may leave oxygen-containing groups, affecting electrical conductivity. Post-Treatment Required: Additional thermal or chemical reduction may be needed to obtain high-quality rGO.
		Modified Hummers	KMnO_4_, H_2_SO_4_, H_3_PO_4_	Eco-Friendly Approach: Eliminates NaNO_3_, reducing the production of hazardous nitrogen oxides (NOx). Higher Oxidation Efficiency: Optimized reagent ratios improve GO yield and oxidation levels. Better Layer Separation: Produces thin-layer GO with improved dispersion and exfoliation. Scalability: Suitable for bulk production, making it viable for industrial applications.	Strong Acid Usage: Still requires high concentrations of H_2_SO_4_, posing corrosive and handling risks. Residual Functional Groups: It may leave oxygen-containing groups, affecting electrical conductivity. Post-Treatment Required: Additional thermal or chemical reduction may be needed to obtain high-quality rGO.
	Exfoliation (graphene synthesis)	Scotch Tape Method	mechanical exfoliation	High-Quality Graphene: Produces single-layer graphene with minimal defects, making it ideal for fundamental research. Simple and Low-Cost: Requires no complex equipment—just graphite and adhesive tape Preserves Intrinsic Properties: Maintains graphene’s electronic and mechanical properties without introducing chemical impurities.	Not Scalable: The process is manual and time-consuming, making it unsuitable for large-scale production. Low Yield: Produces small graphene flakes, limiting its use in industrial applications. Irregular Flake Size: The exfoliated graphene layers vary in size and thickness, requiring additional processing.
		Liquid-Phase Exfoliation (LPE)	sonication	Scalability: LPE allows for large-scale production, making it suitable for industrial applications. Cost-Effective: Compared to CVD, LPE is more economical, as it uses readily available solvents and equipment. Versatile Solvent Choices: Various solvents, including water, ethanol, and NMP (N-methyl-2-pyrrolidone), can be used to optimize graphene dispersion. Solution-Based Processing: Enables easy integration into inks, coatings, and composites, facilitating applications in electronics and energy storage.	Structural Defects: The exfoliation process can introduce defects, affecting electronic and mechanical properties. Limited Control Over Layer Thickness: Unlike CVD, LPE may result in non-uniform graphene layers, requiring post-processing. Solvent Residue Issues: Some solvents may leave residual impurities, affecting graphene quality. Energy Consumption: Ultrasonication requires high energy inputs, which can impact efficiency at larger scales.
		Chemically Reduced GO	reduction of GO	Scalability: Chemical reduction allows for large-scale production, making it suitable for industrial applications. Cost-Effective: Compared to methods like CVD, this approach is more economical. Versatile Reducing Agents: Various chemicals, such as hydrazine, ascorbic acid and sodium borohydride, can be used to tailor graphene properties. Solution-Based Processing: Enables easy dispersion in solvents, facilitating integration into composites and coatings.	Residual Functional Groups: The reduction process often leaves behind oxygen-containing groups, affecting electrical conductivity. Structural Defects: Chemically reduced GO tends to have higher defect density, impacting mechanical and electronic properties. Environmental and Safety Concerns: Some reducing agents, like hydrazine, are toxic and hazardous, requiring careful handling. Incomplete Reduction: Achieving fully reduced graphene is challenging, often requiring additional thermal or electrochemical treatments.
Bottom-Up	Graphene synthesis	Chemical Vapor Deposition (CVD)	carbon gas decomposition	High Purity and Quality: Produces graphene with low defect density, making it ideal for electronic applications. Scalability: Can be used for large-area graphene growth, suitable for industrial production. Precise Control: Allows for fine-tuning of thickness, layer number and crystallinity by adjusting deposition parameters. Versatile Substrates: Works with various substrates, including copper, nickel and silicon carbide, enabling diverse applications.	High Temperature Requirements: Typically requires 900–1100 °C, increasing energy consumption. Complex Setup: Requires specialized equipment and vacuum systems, making it costly. Limited Substrate Compatibility: Some materials may not withstand the high temperatures needed for graphene growth. Transfer Challenges: Graphene grown on metal substrates often requires transfer processes, which can introduce defects.
		Pyrolysis of Organic Precursors	thermal decomposition of organics	Scalability: Pyrolysis allows for large-scale production of graphene, making it suitable for industrial applications. Versatile Precursors: A wide range of organic materials (e.g., polymers, biomass, hydrocarbons) can be used, enabling customized graphene properties. Cost-Effective: Compared to methods like CVD, pyrolysis can be more economical, especially when using biomass-derived precursors. Environmentally Friendly Options: Using biomass waste as a precursor promotes sustainable graphene synthesis.	Structural Defects: Pyrolysis often produces graphene with defects, affecting its electronic and mechanical properties. Limited Control Over Layer Thickness: Unlike CVD, pyrolysis may result in non-uniform graphene layers, requiring post-processing. High Temperatures Required: The process typically operates at 600–1200 °C, increasing energy consumption. Purity Issues: Residual impurities from organic precursors can affect graphene quality, necessitating additional purification steps.
		Epitaxial Growth on SiC	high-temperature sublimation	Direct Growth on a Semiconductor: Graphene forms directly on a semiconducting or semi-insulating substrate, eliminating the need for transfer steps. Large-Area Graphene: The graphene sheet can be as large as the SiC substrate, making it suitable for device fabrication. High Electronic Quality: Produces graphene with low defect density and high carrier mobility, ideal for high-frequency electronics. Stable and Controllable Process: Growth conditions can be optimized to achieve uniform thickness and layer control.	High Temperature Requirement: Requires annealing at temperatures above 1650 °C, which increases energy costs. Limited Scalability: While suitable for research and specialized applications, large-scale industrial production remains challenging. Substrate Influence: The SiC substrate affects graphene’s electronic properties, requiring additional processing steps to modify its behavior. Expensive Material: High-quality SiC substrates are costly, making this method less economically viable compared to other graphene synthesis techniques.

**Table 2:** Fabrication methods for graphene/GO bio-nanocomposites.

Method	Biopolymer–Graphene Nanocomposites	Biopolymer–GO-Based Nanocomposites	Advantages	Disadvantages
Solution intercalation [1]	Graphene is dispersed in a biopolymer solution and cast into films.	GO dispersed in biopolymer solution and reduced to rGO if needed.	Simple, cost-effective and good control over film thickness.	Requires effective dispersion of graphene, solvent evaporation can be slow.
In situ polymerization [2]	Graphene is mixed with monomers or pre-polymers, followed by polymerization.	GO is incorporated and polymer chains graft onto GO.	Stronger interaction between filler and polymer; customizable properties.	A complex process: it requires precise control over reaction conditions.
Melt intercalation [3]	Graphene is mixed with molten biopolymer in an extruder.	GO can be pre-treated and then blended with molten biopolymer.	High throughput; suitable for thermoplastic biopolymers; uniform distribution.	Difficulty dispersing graphene uniformly, need high temperatures.
Solvent evaporation/film formation [86]	Graphene dispersed in biopolymer solution and solvent evaporated to form films.	GO dispersed and then reduced to rGO during film formation.	Simple, easy to scale, good for thin-film fabrication.	Solvent evaporation may be slow, needs effective dispersion of graphene.
Hydrothermal/solvothermal [4]	Graphene and biopolymer treated under high pressure and temperature.	GO reduced in situ during hydrothermal or solvothermal process.	High-quality nanocomposites, uniform dispersion, controlled reduction of GO.	Requires high pressure and temperature, complex setup.
Electrospinning [5]	Graphene mixed with polymer solution and electrospun into nanofibers.	GO dispersed and then electrospun and reduced if needed.	High surface area, customizable fiber properties, good for nanofibers.	High-cost equipment and difficulty in achieving uniform dispersion.
Self-assembly [6]	Graphene is dispersed and assembled through non-covalent interactions.	GO self-assembles with biopolymer; can form layered structures.	Low-cost, no need for solvents, can form complex nanostructures.	Slow process, may be difficult to control for large-scale production.
Layer-by-layer (LbL) assembly [7]	Graphene and biopolymer are alternately deposited to form multilayered films.	GO layers are assembled with biopolymer layers.	High precision, allows for control over nanocomposite architecture.	Requires careful control over layer deposition, can be time-consuming.
Physical blending [8]	Graphene mixed with biopolymer using mechanical methods.	GO physically blended into biopolymer.	Simple, fast, low-cost process, scalable.	Poor dispersion of graphene, potential for poor interface bonding.

**Table 3:** Applications of different GO-PEG composites in tissue engineering.

	Composite	Application	Reference
Bone tissue engineering	PEG-GO-Propylene fumarate	Bone tissue engineering	[204]
	GO-PEGDA hydrogels	Osteogenesis of human adipose-derived stem cells (hADSCs)	[205]
	GO-incorporated PEGDA cryogels	Osteogenic commitment of human tonsil-derived MSCs (hTMSCs)	[206]
Cardiac tissue engineering	GO-PEG hybrid scaffold	Neonatal rat ventricular myocytes (NRVMs)	[207]
Skin tissue engineering	ADM-GO-PEG/Que hybrid scaffolds	Diabetic wounds	[208]

## Data Availability

The original contributions presented in this study are included in the article. Further inquiries can be directed to the corresponding author.
